# To be or not to be vaccinated against COVID-19 – The adolescents’ perspective – A mixed-methods study in Sweden

**DOI:** 10.1016/j.jvacx.2021.100117

**Published:** 2021-10-19

**Authors:** S. Nilsson, J. Mattson, M. Berghammer, A-L. Brorsson, M. Forsner, M. Jenholt Nolbris, I. Kull, A. Lindholm Olinder, S. Ragnarsson, A-C. Rullander, L-L. Rydström, M. Andréia Garcia de Avila, P. Olaya-Contreras

**Affiliations:** a University of Gothenburg, Institute of Health and Care Sciences, and University of Gothenburg Centre for Person-Centred Care (GPCC), Sahlgrenska Academy, Gothenburg, Sweden; b Red Cross University College, Institute of Health Care, Karolinska Institute, Department of Learning, Informatics, Management and Ethics, Stockholm, Sweden; c University West, Department of Health Sciences, Trollhättan, Sweden; d The Queen Silvia Children’s Hospital, Department of Pediatrics, Gothenburg, Sweden; e Karolinska Institute, Department of Neurobiology, Care Sciences and Society, Huddinge, Sweden; f Karolinska Institute, Department of Biosciences and Nutrition, Stockholm, Sweden; g Umeå University, Department of Nursing, Umeå, Sweden; h Karolinska Institute, Department of Clinical Science and Education, Södersjukhuset, Stockholm, Sweden; i Sachs’ Children and Youth Hospital, Stockholm, Sweden; j Umeå University, Institute of Epidemiology and Global Health and Institution of Care Science, Sweden; k Botucatu Medical School - UNESP, Nursing Department, Botucatu, Brazil

**Keywords:** Adolescence, Anxiety, COVID-19, Mixed-methods, Pandemic, Vaccination hesitancy

## Abstract

Vaccination of the population seems to be an important strategy in halting the COVID-19 pandemic in both local and global society. The aim of this study was to explore Swedish adolescents’ willingness to be vaccinated against COVID-19 and its association with sociodemographic and other possible factors. A survey was distributed in Sweden between 7 July and 8 November 2020. The main qualitative question concerned adolescents’ thoughts on vaccination against COVID-19 and evaluated whether the adolescents would like to be vaccinated when a COVID-19 vaccine is made available. In total, 702 adolescents aged between 15 and 19 responded to the questionnaire. A convergent parallel mixed-methods design was used. The results showed that nearly one in three adolescents had not decided if they wanted to get a COVID-19 vaccine, i.e. 30.5%: n = 214. Of the participants 54.3% (n = 381) were willing to be vaccinated. Girls had higher levels of anxiety about the vaccine compared to boys. In addition, high levels of anxiety impacted on the participants’ willingness to be vaccinated. One reason for being undecided about the vaccine was that participants felt they did not know enough about it. Practising social distancing increased willingness to be vaccinated, as reflected in the qualitative results which showed participants wanted to be vaccinated to protect others. The results impart important knowledge to healthcare professionals and contribute to their communication with adolescents about vaccine hesitancy.

## Highlights

• The majority of adolescents are either willing to be vaccinated or ambivalent.
• Adolescents who are willing to be vaccinated have the highest scores for anxiety.
• Boys are more willing to be vaccinated than girls.
• The degree of social distancing increases willingness to be vaccinated.
• The level of knowledge might influence willingness to be vaccinated.

## Introduction

Adolescence is a phase of life in which opportunities to influence one’s health and future patterns of health are established [1]. The period of adolescence is often labelled as the healthiest time of life and indeed, is a period of low mortality [2]. Even if social background is still a risk factor for mortality in Sweden, a decline in death rates has been confirmed for the period 2000 to 2014 [3]. Nevertheless, being healthy during adolescence is necessary to fulfil the developmental step to adulthood, retaining emotional and cognitive abilities for independence and the ability to participate as a member of society. Adolescence is also a critical period in determining health trajectories over the life course. As adolescents become the next generation of parents, they will influence their children’s health habits [2].

The 2019 coronavirus disease (COVID-19) caused by the SARS-CoV-2 first appeared in December of 2019. On 11th March 2020, COVID-19 became a pandemic [4], causing a variety of disease symptoms in the populations of the world. In 2020, over 2 million individuals in Sweden had at least one of six prognostic factors for severe COVID-19, i.e. aged 70 years and older, severe asthma, cancer, cardiovascular disease, chronic obstructive pulmonary disease or diabetes [5]. During the autumn of 2020, Sweden had one of the highest numbers of COVID-19 deaths per inhabitants globally [6]. It was shown that adolescents appeared to have a milder disease course and better prognosis than adults [7].

Vaccination of the population seems to be one of the most important strategies for halting the pandemic both locally and globally. Consequently, as early as nine months after the beginning of the COVID-19 outbreak, preclinical and early clinical data were available on vaccines [8] and rapid development of new vaccines was achieved. Such rapid development and production are not new. A decade ago, populations in Sweden and other parts of the world were vaccinated during the H1N1 influenza (Swine flu) pandemic. However, side effects of the vaccine were seen among children and adolescents [9]. Some years after the H1N1 vaccination programme, mistrust of a new vaccine and its safety were categories of concern among potential recipients of the Human Papilloma Virus (HPV) vaccine in Europe [10] and parents in Sweden [11].

Adolescents have a role to play in achieving herd immunity in society. As schools can be an important source of ongoing transmission and outbreaks of COVID-19, the vaccination of adolescents seems to be necessary in order to stop transmission of the disease [12].

In Sweden, there is a high level of trust in authorities and medical science and therefore vaccination rates are often high. However, after the incidence of narcolepsy in conjunction with the H1N1 vaccination, scepticism to vaccination has increased, and this may affect the vaccination rates for influenza vaccinations, new vaccinations, and relatively safe and evidence-based measles vaccinations [13]. Vaccine hesitancy is also a challenge in the COVID-19 pandemic [14], with social media playing an increasing role in the spread of vaccination hesitancy [15]. Among adults who were reluctant to receive a COVID-19 vaccine, social media is reported as being an important influence, especially if these individuals mistrusted and were less likely to obtain information about the pandemic from traditional and authoritative sources [16].

In Europe, adults’ willingness to be vaccinated against COVID-19 has mainly been influenced by fear of side effects [17], [18]. Although the predicament of adolescents during the COVID-19 pandemic has been previously described [19], to our knowledge there has been no prior investigation of their willingness to be vaccinated. How adolescents consider vaccination is not well studied and it is unclear how adolescents in Sweden argue their choice regarding vaccination, when this becomes available to them.

The aim of this study was to explore Swedish adolescents’ willingness to be vaccinated against COVID-19 and its association with sociodemographic and other possible factors.

## Methods

### Study design

An observational study was performed to reveal the association between willingness to be vaccinated for COVID-19 and demographic and additional factors in a group of adolescents from Sweden. A convergent parallel mixed-methods design was used [20].

### Participants

In total, 702 adolescents aged 15–19 participated in this study (Table 1). The majority live in good social conditions, i.e. live in a detached house (58.3%), in middle and small-sized towns (90.1%), and together with family members between 4 and 7 persons (67.1%).

**Table 1:** Characteristics of the adolescents.

Total *n* = 702	*n*	%
Age	671	
15–17 years	502	71.5
18–19 years	169	24.1
Sex	700	
Boys	296	42.2
Girls	404	57.5
Size of town	702	
≥500 000 inhabitants	69	9.8
100 000–499 999 inhabitants	326	46.4
0–99 999 inhabitants	307	43.7
Type of abode	681	
Detached house	409	58.3
Town house	101	14.4
Apartment	171	24.4
No. people in the same household	701	
1–3 persons	210	29.9
4–7 persons	471	67.1
≥8 persons	20	2.8
Education	698	
High school	14	2.0
Upper secondary school	651	92.7
Programme in upper secondary school	629	
Academically oriented programmes	475	67.7
Vocational programmes	130	18.5

### The questionnaire

The questionnaire (containing 20 items) has previously been used in Brazil [21], and was adapted to a Swedish context on the web platform esMakerNX3 - V 3.0. The questionnaire captured data regarding sociodemographics, the adolescents’ schooling, and social distancing in relation to COVID-19. The Numerical Rating Scale (range 0–10) (NRS) was also used to assess anxiety [22]. This study probed two additional questions, the first being the open question: *Feel free to comment about your thoughts on vaccination against COVID-19,* on which the qualitative data was based. The second question: *Would you like to be vaccinated when a COVID-19 vaccine becomes available?* was the basis for the quantitative data. The questionnaire took approximately 5–10 min to complete.

### Data collection

The adapted version of the questionnaire was tested in a pilot study with 13 participants. These data were not included in the main data collection. After the pilot study, a convenience sampling method through snowballing was conducted between 7 July and 8 November 2020 in Sweden. The survey was distributed by all the Swedish authors, through social media (Facebook, Instagram), schools, sports clubs, youth camps and scout associations. A brief written description of the study and its objectives was included in the beginning of the survey.

### Qualitative analyses

The qualitative data were analysed from the answers to the open question *Feel free to comment about your thoughts on vaccination against COVID-19.* Existing knowledge of the phenomena was deemed scarce and fragmented, leading to an inductive content analysis according to Elo and Kyngäs [23] as described below. Data were exported to NVivo 12 Pro and read several times before commencing the coding of words. In the inductive content analysis, the words are distilled into content-related categories. According to Elo and Kyngäs [23] it is assumed that “when classified into the same categories, words, phrases and the like share a common meaning”. Each of the answers were seen as a unit of meaning. The coding was performed manually with the help of NVivo 12 Pro, which suggested preliminary categories. These categories were then collated on to coding sheets and abstracted by dividing them into sub-categories. Generic categories were created as the categories were abstracted further.

### Quantitative analyses

Willingness to be vaccinated i.e. *Would you like to be vaccinated when the vaccine becomes available?* was defined as the dichotomous dependent variable, organised as follows: 1: Adolescents rejecting vaccination or unsure; 2: Adolescents willing to be vaccinated. Descriptive characteristics of the ordinal variables and by vaccine willingness were computed by the Kruskal-Wallis tests and the Independent-Samples Median test (Yate’s Continuity Corrected Asymptotic Sig, and Bonferroni correction for multiple tests). The Chi-Square test, as a two-tailed test (*n* > 30), and the Fisher-exact test were employed to compare proportions in the different groups. A total of 702 adolescents were included in the analyses. The NRS scores (0–10) were categorized using the NRS cut-off > 6 (percentile 75), [24]. Missing values were not included in the statistical analysis.

The measures of association between the factors studied and the willingness of vaccination were expressed by the Odds Ratio (OR) with a 95% confidence interval, using multivariate logistic regression. The first category of the dependent variable (Yes) was taken as the baseline category, and the results were interpreted accordingly. All the independent variables, i.e. sociodemographic and variables towards the pandemic were tested in the univariate analyses. The contribution of each variable to the model was examined by the Likelihood ratio-test (χ2, p < 0.05). Multiple models were tested using the Stepwise analyses. Goodness of fit of each model was tested with the deviance coefficient and the Hosmer-Lemeshow statistic. The final model was adjusted for programs in upper secondary school (academically oriented programs/vocational), assessed as the main confounder (n = 73/702 missing values), place of residence (big, medium sized or small town), and for the number of persons living in the same house. The interaction between gender and levels of anxiety was tested by multiple models. For all tests, the level of statistical significance was set at 5%. For the statistical analyses, we used IBM SPSS Statistics, version 27 (IBM Corp., Armonk, N.Y., USA).

### Ethical aspects

The survey was anonymous, and all participants received information about the study. Ethical approval was obtained from the Swedish Ethical Review Authority (ref 2020-02547).

## Results

### Qualitative results

The answers to the open-ended question *Feel free to comment about your thoughts on vaccination against COVID-19* gave a broadness in positioning among the adolescents, from a clear ‘yes’ to a more deliberated approach. Three generic categories emerged through the analysis: *The adolescents expressed a need to know more*, *The adolescents did not consider themselves to be in need of vaccination* and *The adolescents expressed a willingness to be vaccinated for the sake of others*, are presented below.

#### The adolescents expressed a need to know more

The adolescents’ answers demonstrated that they had reflected on how to decide by weighing the pros and cons and risks of vaccination versus the benefits. Although the participants described a willingness to be vaccinated and did not want to be negative towards it, they wanted to know more. For example, they raised the need to further research the procedure, as its rapidity made them uncertain. The risk of adverse events was a particular concern, with reference to narcolepsy, which occurred after the mass vaccination against the H1N1 influenza (Swine flu).

*I would get vaccinated if proper tests have been done – so it’s not like that influenza vaccine that could bring on narcolepsy.*

Another argument against vaccination was the fear of injections:

*I have a needle phobia and I’m scared of the bad sides of a vaccine that haven’t been found yet but that people discover after the vaccination has been done.*

#### The adolescents did not consider themselves to be in need of vaccination

The youths said they were healthy and not at all at risk. They did not consider vaccination necessary because COVID-19 was not ‘their disease’, meaning they were not as susceptible to infections and would not be affected by or die from the disease.

They did not fear COVID-19 as death rates are low in their age group and some said they would rather be infected than vaccinated.

*I feel it’s better to be infected when it isn’t dangerous for me to be infected.*

#### The adolescents expressed a willingness to be vaccinated for the sake of others

The adolescents conveyed an altruistic perspective, in that they felt a general responsibility towards society was a reason for being vaccinated. It was seen as important to protect the elderly and people in risk groups, but also to benefit the economy. Moreover, by being vaccinated themselves, they could contribute to herd immunity, which in turn would protect those who could not be vaccinated.

*I think a vaccine against COVID-19 is good because it protects us from getting sick, which leads to society being able to open up and older people don’t need to live as isolated as they are now, barely able to go out and with lots of restrictions they have to follow so they don’t risk getting sick.*

### Quantitative results

Answering to the question *Would you like to be vaccinated when the vaccine becomes available?* Overall, more than half of the adolescents 54.3% (*n* = 381/702) answered yes, 15.2% (*n* = 107) no, and 30.5% (*n* = 214) unsure (Table 2).

**Table 2:** Factors associated with willingness to be vaccinated.

*Willingness to be vaccinated when the vaccine becomes available* *(Total values for each variable)*	*Yes* *(n = 381/702)*^1^*54.3%*	*No/Undecided* *(n = 321/702)*^1^*45.7%*	_Adj_OR (*95% CI*) 2(n = 601) 2
	(n)%	(n)%	
*Gender*^^*^ Boys (2 9 6) 42.3% Girls (4 0 4) 57.7%	(1 7 9) 25.6 (2 0 0) 28.6	(1 1 7) 16.7 (2 0 4) 29.1	1 0.61 (0.43 – 0.86)^1^
Total: (7 0 0)*^+^* 100%	(3 7 9) 54.1	(3 2 1) 45.9	
*Refrain from social activities or group training during the pandemic*^^*^ Yes (4 6 0) 65.8% Not at all (2 3 9) 34.2%	(2 6 2) 37.5 (1 1 7) 16.7	(1 9 8) 28.3 (1 2 2) 17.5	1 0.64 (0.45– 0.91) ^1^
Total: (6 9 9)*^+^* 100%	(3 7 9) 54.2	(3 2 0) 45.8	
Living with someone with COVID-19^**^ Yes (88) 12.5% No/Don’t know (6 1 4) 87.5%	(41) 5.8 (3 4 0) 48.4	(47) 6.7 (2 7 4) 39.0	1 1.99 (1.20 – 3.34) ^1^
Total: (7 0 2) 100%	(3 8 1) 54.3	(3 2 1) 45.7	
*NRS* S*cores*^^**^ NRS ≤ 6 (5 7 3) 85.3% NRS > 6 (99) 14.7%	(3 0 4) 45.2 (68) 10.1	(2 6 9) 40.0 (31) 4.6	1 2.09 (1.26 – 3.45) 2
Total: (6 7 2)*^+^* 100%	(3 7 2) 55.4	(3 0 0) 44.6	

Legend: *^+^* Missing values were not included in the model (NRS n = 30; sex n = 2, Refrain from social activities n = 3). ^1^For the descriptive analyses ^Pearson Chi-Square, *Fisher Exact Test, and **McNemar Test were performed, sig *p < 0.05.

2 Results from the logistic regression: For the dependent variable “Reject being vaccinated”, ‘No/unsure’ was used as the reference category. The first category of the independent variables was considered as the reference (1). ^2^**Model**: **(n = 601)** gender, refrain from social and group activities, having anyone in the family with Covid-19, Adjusted by type of education (^+^Academically oriented/Vocational, n = 629/702), place of residence (large, medium-sized or small town), and for number of persons living in the same house. *p < 0.01. Missing values were not included in the analyses.

Among the participants saying yes to vaccination, 52.8% were girls (*n* = 200/379) and 47.2% were boys (*n* = 179; 2 missing values), while among the adolescents rejecting, 63.6% were girls (n = 204/321) and 36.4% were boys (n = 117; n = 701, p = 0.004).

In the multivariate models, four of the independent variables (gender, living with someone infected by COVID-19, degree of refraining from social activities, and NRS scores) remained statistically significantly associated with the adolescent’s willingness to be vaccinated after controlling for confounding factors (Table 2). Whereas variables regarding the pandemic, i.e. attending school during the pandemic, receiving distance education, social isolation during the pandemic, and comprehension of the current pandemic situation affecting society were not statistically significantly associated with willingness to be vaccinated (Data not shown).

The probability of the girls being willing to be vaccinated (say yes) was 39% less than the boys (_Adj_OR = 0.61, CI 0.43–0.86; Table 2).

Of all the participants, 65.8% (*n* = 460/699) had refrained from social activities and group training. For the group saying yes to vaccination, 30.9% had not refrained (*n* = 117/379) compared to 69.1% (*n* = 262) who had refrained from social and group training; p = 0.04). The participants who had not refrained from their normal social activities or group training, i.e. remained in contact with peers, were 36% less likely to say yes to vaccination than participants who refrained (_Adj_OR = 0.64, CI 0.45–0.91; Table 2).

Among the participants saying yes to vaccination, 89.2% (*n* = 340/381) had not lived with someone who was infected with COVID-19, and 10.8% (*n* = 41) did; p < 0.05). Adolescents who were not living with someone infected by COVID-19 were almost twice as likely to say yes to vaccination compared to those living with someone infected (_Adj_OR = 1.99, CI 1.2–3.35).

#### The association between willingness to be vaccinated and anxiety scores

The percentile 75 corresponded to NRS = 6. Of the adolescents, 14.7 % (*n* = 99/672) exhibited the highest NRS scores (NRS > 6). When using a cut-off NRS > 7, the prevalence of anxiety was 5.3%. Among the group willing to be vaccinated, 81.7% (*n* = 304/372) had lower NRS scores (NRS ≤ 6) and 22.7% (*n* = 68/300) had higher scores on anxiety (NRS > 6). In contrast, among the participants who rejected vaccination, 89.7% (*n* = 269/300) had lower NRS scores (NRS ≤ 6) and 10.3% (*n* = 31) had higher scores (NRS > 6, p = 0.004; Table 2).

**Fig. 1:**
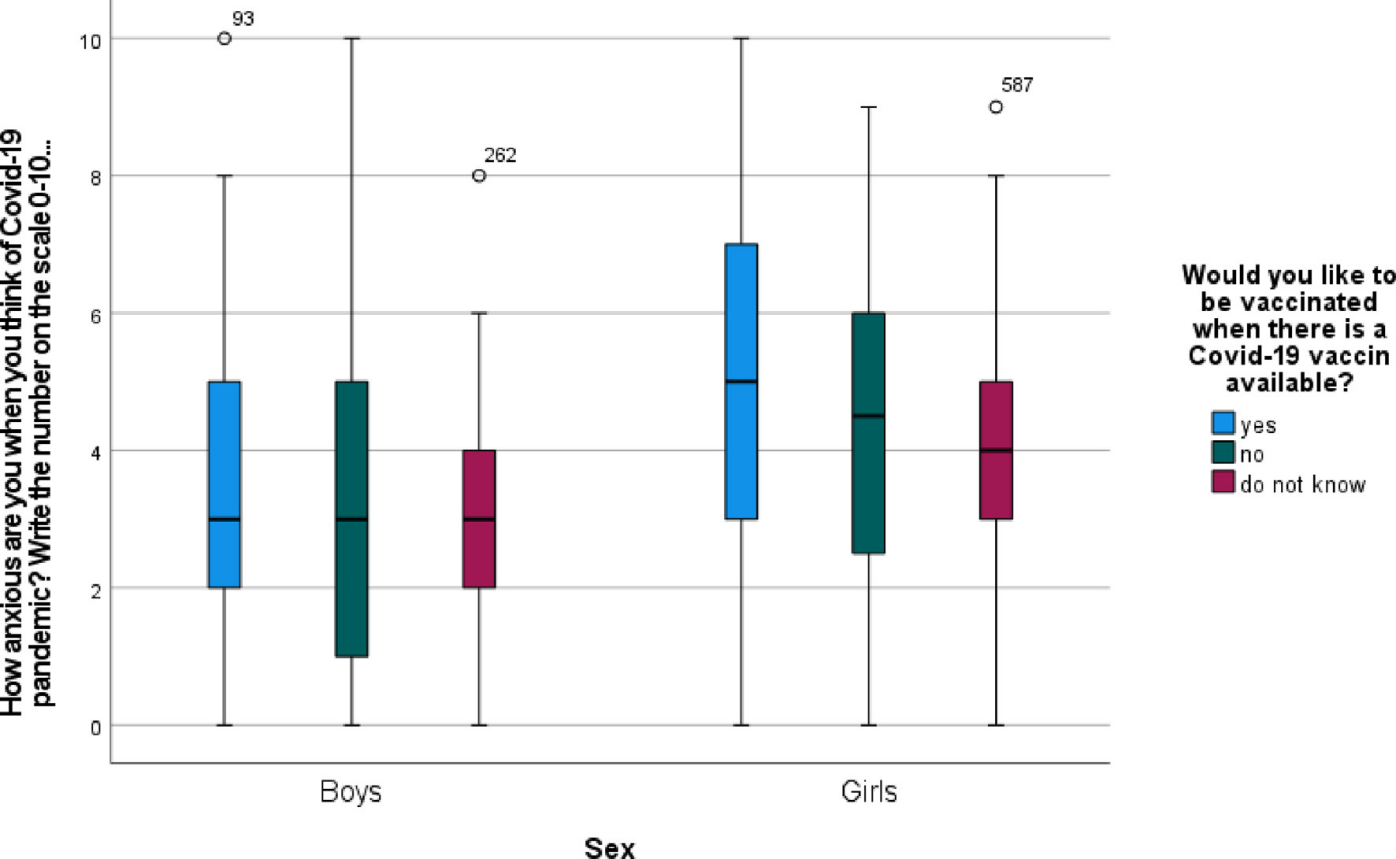
Boxplots with median values of the NRS scores for girls and boys, by willingness of vaccination (yes, no, undecided). Independent-Samples Median-Test and the Bonferroni correction for multiple tests p < 0.001; (n = 670, 2 missing values on gender).

Overall and in all groups, the girls exhibited higher median scores for NRS (Md = 5.0), especially among girls who said yes to vaccination compared to the boys (Md = 3.0; p < 0.001; *n* = 670; Fig. 1). Both the whole group saying yes to vaccination and the girls rejecting vaccination had Md = 4 (Fig. 1).

Additionally, the adolescents who said yes to vaccination and refrained from social activities or group training exhibited the highest median values of NRS compared to adolescents who rejected vaccination (median yes = 4.47/, unsure = 4.11; p < 0.010).

The adolescents with higher scores for NRS (NRS > 6) were twice as likely to get vaccinated (_Adj_OR = 2.09, CI 1.26–3.45) than those with lower scores for NRS (NRS ≤ 6; Table 2).

### Integration of qualitative and quantitative results

**Fig. 2:**
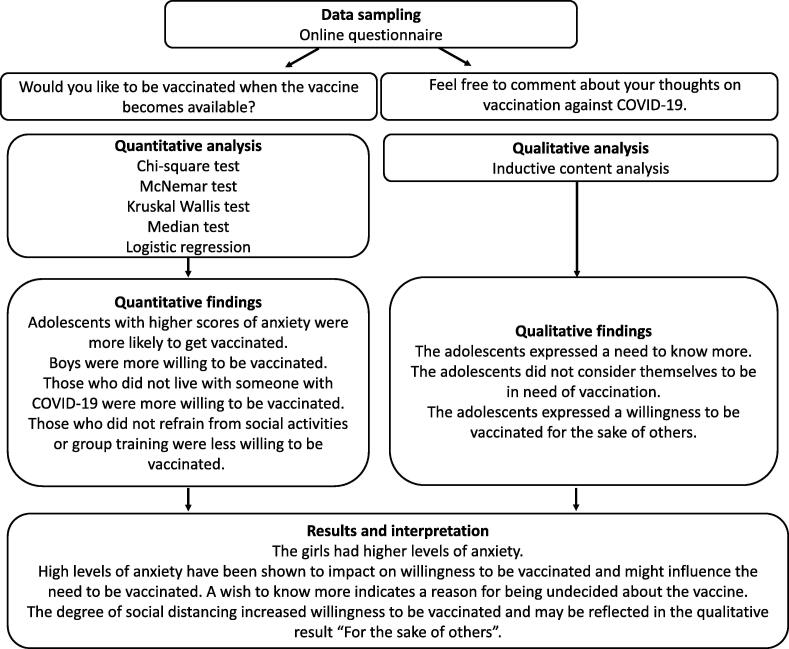
Integration of qualitative and quantitative results.

Fig. 2 shows the integration of our qualitative and quantitative results. Levels of anxiety were found to impact on the willingness to be vaccinated and might reflect the qualitative finding that youths did not consider themselves to be in need of a vaccination. There was uncertainty about whether or not to have the vaccine and a wish to know more about the pros and cons of doing so. Refraining from social activities and the degree of social distancing seemed to increase willingness to be vaccinated and might reflect the qualitative finding that youths are willing to be vaccinated for the sake of others (Fig. 2).

## Discussion

The results of this study show that quite a number had not yet decided about getting a COVID-19 vaccine – almost a third (30.5%: n = 214) of the adolescents. Afifi, Salmon [25] found similar percentages of willingness, i.e. 26.1% of the adolescents and young adults in Canada were undecided about getting a COVID-19 vaccine. However, 65.4% of these reported willingness to be vaccinated. In this study, 54.3% (n = 381) of the adolescents were willing to be vaccinated. Assumptions about the level of vaccination rate to achieve herd immunity vary, but the threshold for SARS-CoV-2 has been suggested to be between 50% and 67% [26]. Consequently, it is necessary for more of the undecided adolescents to be willing to get a vaccination. Vaccination hesitancy has been an obstacle among adolescents, and several strategies have been implemented with the aim of improving vaccination rate, for example, health education, financial incentives, mandatory vaccination, and class-based school vaccine delivery. However, the evidence for those interventions is still to be evaluated [27], [28].

This study highlights that knowledge about the situation influences willingness to be vaccinated. These findings are in line with a review reporting that information about HPV vaccination increased the vaccination rate in adolescents [27]. Adolescents who had higher scores in knowledge about HPV exhibited a higher vaccination rate [29]. In the COVID-19 pandemic, a study among adults in the USA confirmed that vaccination hesitancy is influenced by conspiratorial disinformation about vaccines [30].

There exists a complexity in understanding what influences vaccination acceptance in a population. The 5C is an attempt to describe psychological constructs which explain this. The 5C evaluates: 1) *Confidence* – referring to trust in the vaccine, the system of delivery and the need for a vaccine. 2) *Complacency* – referring to the perceived risks of vaccine-preventable diseases. 3) *Constraints* – referring to the availability, accessibility and affordability of the vaccination. 4) *Calculation* – referring to calculating the balance of risks of infections and the risks of a vaccination. 5) *Collective responsibility* – referring to the willingness to protect others by herd immunity [31]. The results of this study support that 5C can be used in explaining the willingness of adolescents to be vaccinated, i.e. both the qualitative and quantitative data in this study can be explained by using the 5C. Anxiety surrounding COVID-19 and uncertainty regarding the safety of the vaccine influenced the adolescents’ willingness to be vaccinated. However, willingness to protect others also influenced their decisions.

During this pandemic, the Swedish media (and that of other countries) has highlighted adolescents’ selfishness and non-compliant behaviour [32]. However, this study has revealed adolescents demonstrate altruistic motives for being vaccinated – namely to protect others from COVID-19. This can be linked to a step in Gilligan’s theory on moral development, from selfishness to responsibility to others [33], and is in line with previously reported findings that when some people are unable to get vaccinated, the willingness of others to be vaccinated increases in order to indirectly protect them [34].

The quantitative results show the adolescents were more likely to get a vaccination if they did not have a COVID-19 infected person in their household. One explanation for this could be that adolescents who did have an infected person in their household believed they had gained natural immunity and were consequently less afraid of exposure to the virus.

In this study, the boys were more willing to be vaccinated in all cases. This is in line with the adult population in another study, where male gender was associated with a higher potential to accept COVID-19 vaccination [35]. However, among adolescents and young adults in Canada, willingness was not influenced by age, sex or mental health conditions but did differ in relation to social/physical distancing, which is in line with our findings about social distancing [25].

In our study, levels of anxiety regarding COVID-19 were generally low. A noticeable result from our data is that most of the adolescents did not report anxiety due to COVID-19. In our sample, the percentile 75 corresponded to NRS = 6. This contrasts with Brazil, where the percentile 75 corresponded to NRS = 7 [21]. Hence, Brazilian children exhibited higher NRS scores than the Swedish adolescents. Thus, the prevalence of anxiety in Swedish adolescents was 14.7% for NRS > 6, and of 5.7% comparing these studies using a cut-off for NRS of NRS > 7, compared to 21.8% for Brazilian children [21]. However, there were differences between these data samples, as the Brazilian children were younger than the Swedish adolescents [21] and Brazilian schools had closed, whereas in Sweden schools were still open [36].

Adolescents with higher scores for anxiety were twice as likely to get vaccinated. However, the girls had higher median NRS values compared to the boys, and girls who said yes to vaccination exhibited the highest median NRS scores. The adolescents’ level of anxiety seemed to influence their willingness to be vaccinated for COVID-19. This is in line with another study, where fear about COVID-19 was associated with willingness to get a COVID-19 vaccine [35].

The results highlighted adolescents’ thoughts about vaccine safety, and that the disease COVID-19 influenced their willingness to be vaccinated (Fig. 2). Parents or guardians probably influence their adolescent’s willingness to be vaccinated. Bell, Clarke [37] recruited parents or guardians of a child (or children) aged 18 months or under. About half of them were willing to vaccinate themselves (55.8%: n = 699) and to vaccinate their children (48.2%: n = 604). In another study, caregivers of older children (md 7.5 years) were willing to a higher degree (65.2%: n = 1005) to vaccinate their child against COVID-19 [38]. The same parents or guardians of younger children (18 months or under) were also undecided (40.5%: n = 507) about whether they wanted a COVID-19 vaccine themselves and whether they would give the vaccine to their child/children (48.3%: n = 605) [37]. A common reason for declining vaccination was concern about vaccine safety [37], [38].

COVID-19 has not caused a high frequency of death among adolescents. Until November 2020, no deaths related to COVID-19 were reported in Sweden among adolescents [39]. Thus, the perceived risks of a disease influence willingness to be vaccinated. In that sense, a new vaccine might be experienced as a higher risk than the disease itself. Vaccine hesitancy is growing in society, and vaccination coverage is likely to decrease as a consequence of this [40]. In Japan, vaccine coverage declined rapidly from > 70 % to < 1% after unconfirmed reports of adverse events following HPV vaccination. The vaccine crisis was predicted by a modelling to result in around 5,000 deaths from cervical cancer [41]. During the COVID-19 pandemic, adults in the U.S reported that vaccine-related attributes (e.g. vaccine efficacy, adverse effects, and protection duration) were important in their choice of whether or not to be vaccinated [42]. In China, a study reported similar arguments for vaccine rejection among adults, i.e. concerns about side effects and vaccine efficacy [43]. Concerns about safety of the vaccine probably influenced the adolescents in this study. The qualitative results stressed the risk of adverse events, with reference to narcolepsy, which occurred after the mass vaccination against the H1N1 influenza (Swine flu).

Our sample is unique and reflects the opinions of a homogenous group of adolescents coming from medium-sized and small towns and to some extent those living in larger towns. Most of our participants came from typical Swedish families with a good standard of living; thus, our findings reflect the opinions of a specific group coming from very similar socioeconomic conditions. Most of these were girls living with both parents, with a high degree of education, and good finances, living in a house, and with the majority of the parents possibly working in a large town (Table 1). The standard of living that these adolescents enjoy may partially explain the low prevalence of anxiety in our sample.

This study has some limitations. The main limitation is that the data collection was affected by participant selection bias. For instance, our study population was probably less diverse in including adolescents with parents coming from other countries. The data collection involved snowballing, which did not make it possible to determine drop outs. Caution should therefore be taken in generalising the results. A second limitation is the small number of predictors in the final model, although we have controlled for important confounding factors. A third limitation is the date for the data collection and the analysis, as the situation could have changed with the authorization of mRNA vaccines in adolescents. A fourth limitation is that other surrounding factors may affect anxiety and well-being in adolescents, which may affect how they answer the questions.

## Conclusion

The majority of the adolescents in this study were either willing to be vaccinated or ambivalent. Such ambivalence might contribute to reduced uptake of the COVID-19 vaccine. Gender differences in attitudes might also be a factor to consider when the time comes to vaccinate the adolescent population. Levels of anxiety, concerns about the safety of the vaccine, the importance of continuing with physical and social activities, and the potential to protect others influenced the adolescents’ attitudes to getting vaccinated against COVID-19.

## Implications and Contribution

In Sweden, the majority of adolescents are either willing to be vaccinated or ambivalent. Being male with low self-reported anxiety but refraining from social activities and group training might positively influence willingness to be vaccinated.

## Declaration of Competing Interest

The authors declare that they have no known competing financial interests or personal relationships that could have appeared to influence the work reported in this paper.
